# Development of a monitoring scheme for preventive maintenance of the cement machinery

**DOI:** 10.1186/s40064-016-1791-7

**Published:** 2016-03-01

**Authors:** Malek Alhajjaji, Brahim Boubeker, Safia Eljoumani, Abdellah Zamma, Mohamed Idiri, Hafsa Atik

**Affiliations:** Laboratory of Engineering and Materials (LIMAT), Faculty of Science Ben M’Sik, University Hassan II, Casablanca, Casablanca, Morocco; Laboratory of Industrial Engineering, Energy and Sustainable Development (LPEDD) ESTF School of Technology, University Sidi Mohamed Ben Abdellah, Fez, Morocco

**Keywords:** Rotary kiln, Roller shaft, Fatigue, Preventive maintenance, Non destructive testing, FEM

## Abstract

It was underscored that the optimization of policies of preventive maintenance has become a subject of much research. This article proposes a new optimal policy of preventive maintenance for the roller shaft system. It is divided into two main sections. The first proposes a new design of the shaft where the effect of preventive maintenance is integrated, and the second is developing a new control technique adapted to the new design. In this regard, we are interested in the shaft of rollers of the rotary kilns of cement. We have noted the stresses imposed to the axis of the roller. These constraints that have stemmed from the contact between tire and its support rollers. Currently the ultrasonic inspection method of the solid shaft in this situation poses disadvantages, for example, the obligation to stop the rotary Kiln and the difficulty of detecting and sizing defects (fatigue cracks). We propose in this study another approach, we recommend opting for a hollow shaft instead of a solid shaft with a minimum diameter that allows the control system to enter hollow shaft to show for inspection purposes. This will allow preventive control, in operation by the non-destructive technique of ultrasound. The analysis shows the different results of comparison between the axis of full and hollow cylinders, having the same dimensions and the same material and ultimately the most interesting is the same operation.

## Background

The objective in all industry feature the machines and systems need to increasingly efficient is taking consideration the complex of these structure but the requirements of the security, cost optimization and control of equipment availability makes preventive maintenance in the first of all search work. It must allow to intervene only in the presence of defective elements, minimize repair time and to provide a reliable and easily interpretable diagnosis despite the complexity of the equipment (Claire 2002; FLSmidth Institute 2005).

Although our interest will mainly focus on constitutes a significant component in the maintenance. The rotary kiln is used for the pre-treatment step in the production of Portland and other types of cements.

For the rotary kiln, the roller shafts are among the main parts that make up the kiln. They are considered the most important items related to safety because a roller shaft that has damage can directly cause an accident (Chapman 1985).

To minimize processing time, the maintenance method adopted is that of preventive maintenance with a new design of the roller shaft minimizing its weight to facilitate preventive maintenance. Then, a comparison is made between the current shaft and the one developed by our study using the finite element method (FEM) to know the difference between the two shaft reliability.

## Kiln

### Cement rotary kiln

**Fig. 1:**
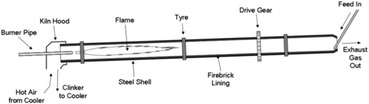
Rotary kiln (Žiga et al. 2008)

The kiln is a pipe rests on supporting station rollers (two, three, eight..) having an inclination of the order of 3.5 % with respect to the horizontal and rotating at speeds between 1.8 and 3.5 r/min (see Fig. 1).

**Fig. 2:**
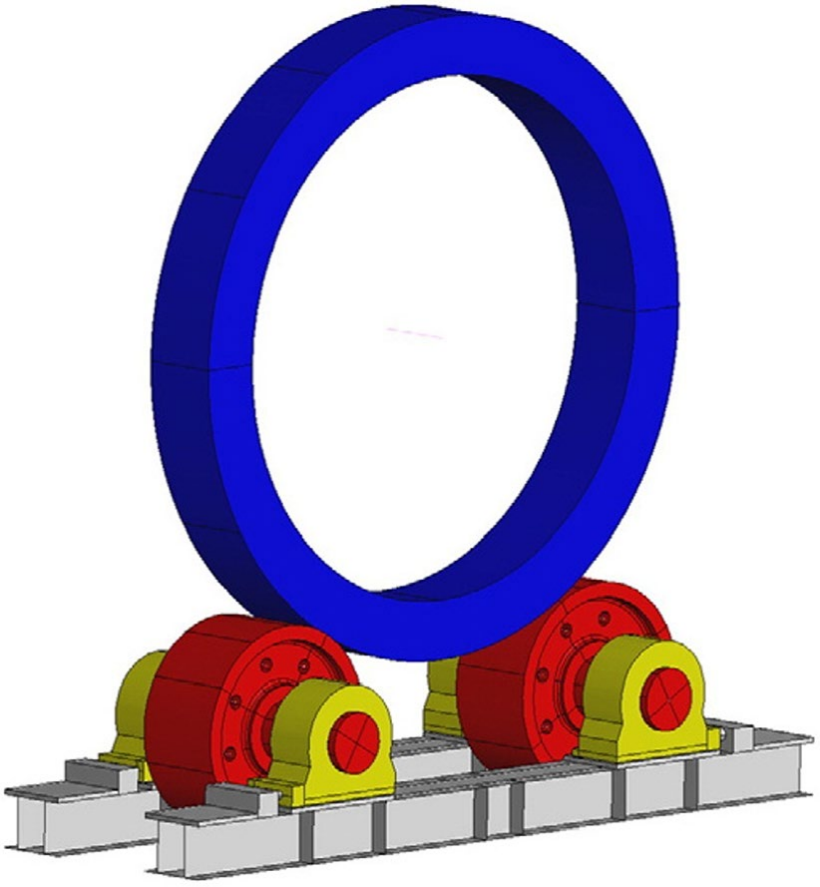
Supporting station assembly (tire & rollers) (Rusinski et al. 2013)

The rotary kiln is isostatic supported by three rolling stations with the upstream stations is a drive roller. The kiln shell is supported via notched tires on the rollers (Fig. 2), rollers are aligned on the tires, the kiln rotation can be ensured in this case by the ring gear/pinion attached to the shell. The movement will be guided by rolling stations.

The kiln is subjected to stresses originating from the mechanical loads implemented or applied, the thermal load of the geometry defects. Thus, the charges of the shell are transmitted to the foundation via the tires, rollers, and bearings (Chapman 1985; Cement kiln 2010).

### Rollers operating principle

The kiln is placed on a set of three tires and six rollers. The tires are weakly attached to the kiln shell. The rotation of the latter is made of three pairs of steel rollers. These rollers must support the oven and allow the rotation to be done, also as possible frictionless.

Support rollers are forged steel compound to shafts Forged carbon steel, and they are equipped with two plain bearings suitable for low-speed machines with high loads and are characterized by good “elasticity” which compensates for changes static aligned in the oven (Polysius 2004; Rusinski et al. 2013).

## Progress study

### The case study presentations

The objective is to optimize the time preventive maintenance of the roller shaft, we reduce the time, material and effort provides for this operation.

Problematic: During operation of the rotary kiln, a reverse bending phenomenon occurs at the roller shaft, which gives rise to fatigue microcracks whose propagation is often the cause of fractures to the roller shaft.

A number of shaft failures have been investigated in detail in recent years, and the results have several common features which are worth enumerating:

- The fracture always has the appearance of a fatigue failure because of the characteristic lines from the point of the initial crack followed by parallel failure lines similar to the growth rings on a tree. There is always a relatively small brittle failure area at the centre of the shaft where final fracture takes place.
- The initial crack usually follows a line at 90 degrees to the shaft axis which indicates that the direction of the primary stress is due to bending of the shaft and not to torsion.
- The initial stress raiser is seldom evident because of subsequent surface damage in the vicinity of the fracture. However, in most cases the evidence suggests the following stress raisers to be responsible (Reid 1988):
  Fretting and pitting corrosion
  Surface defects such as welding inclusions
  Deep machining marks or scratches
  Poor blending of fillet radius into journal
  Wear grooves at or close to fillet radius

**Fig. 3:**
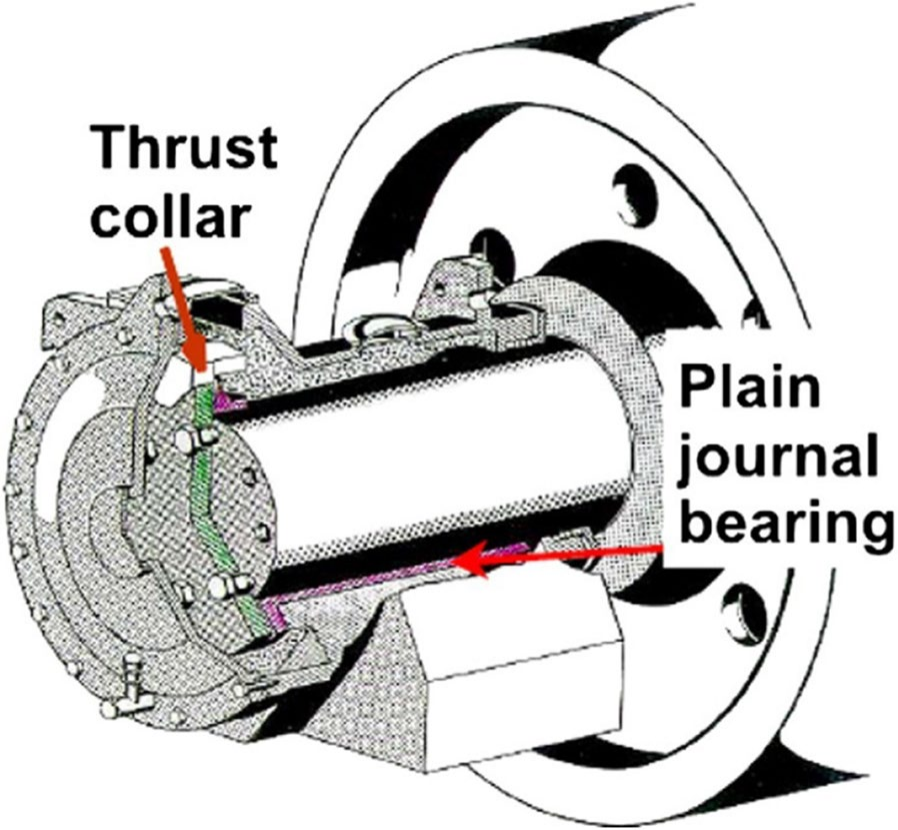
Kiln roller bearings assembly (Polysius 2004)

**Fig. 4:**
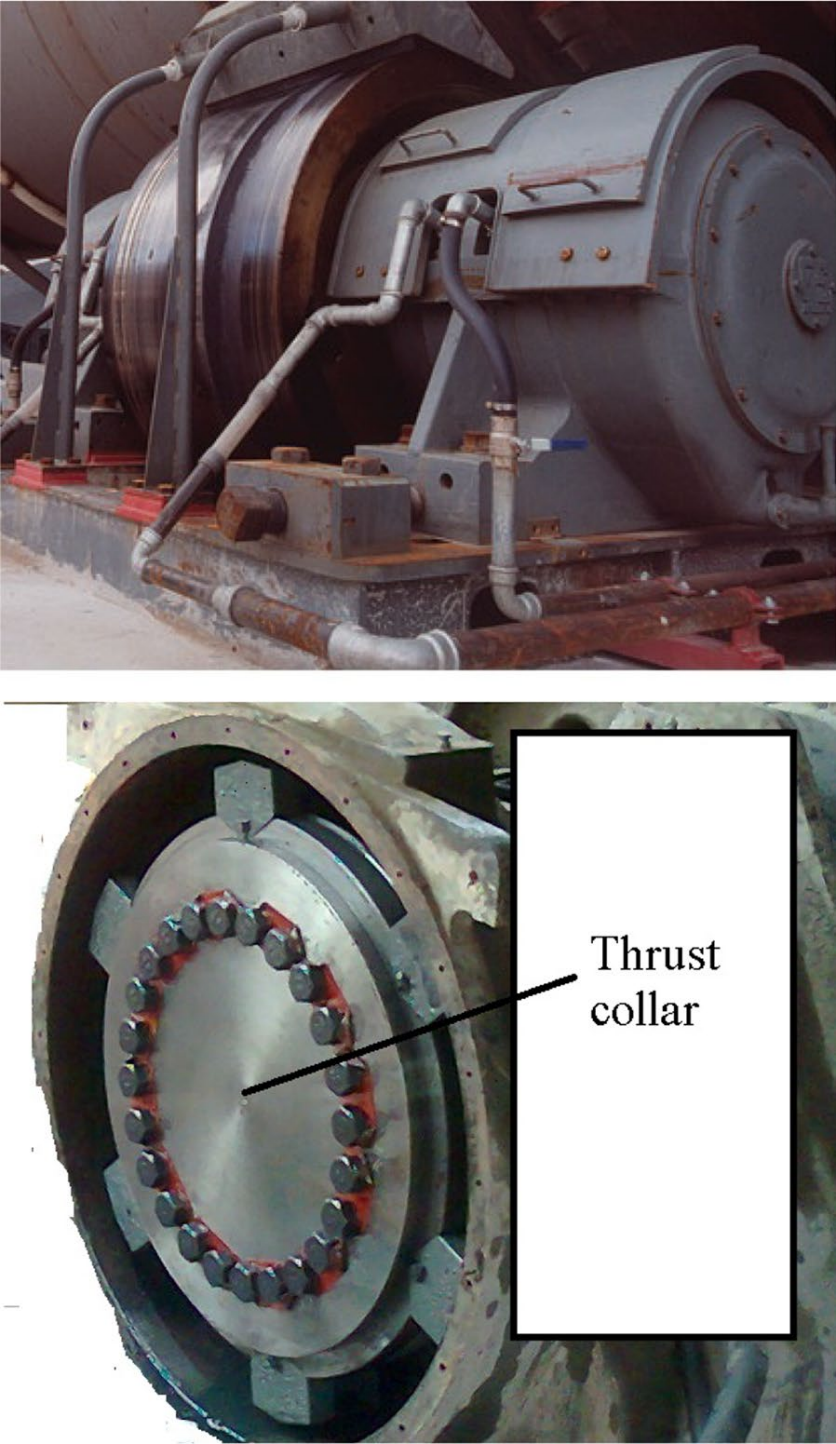
The installation of the roller shaft

Preventive maintenance of the roller shaft by a non-destructive testing technique is hampered by the inaccessibility of these organs targeted by the inspection. The operation requires a significant time to dismantle the covers, thrust collar and the oil change for the six rollers and also for their assembly. Moreover, despite the difficulty in dismantling, we do not get to a result of reliable and precise control because of the complexity of the installation (see Figs. 3, 4).

The purpose of the Research: New methodology and design of a control system for monitoring the rollers shaft and provides on-line information pointing to potential failure.

### Static analysis

#### Data and hypothesis

**Fig. 5:**
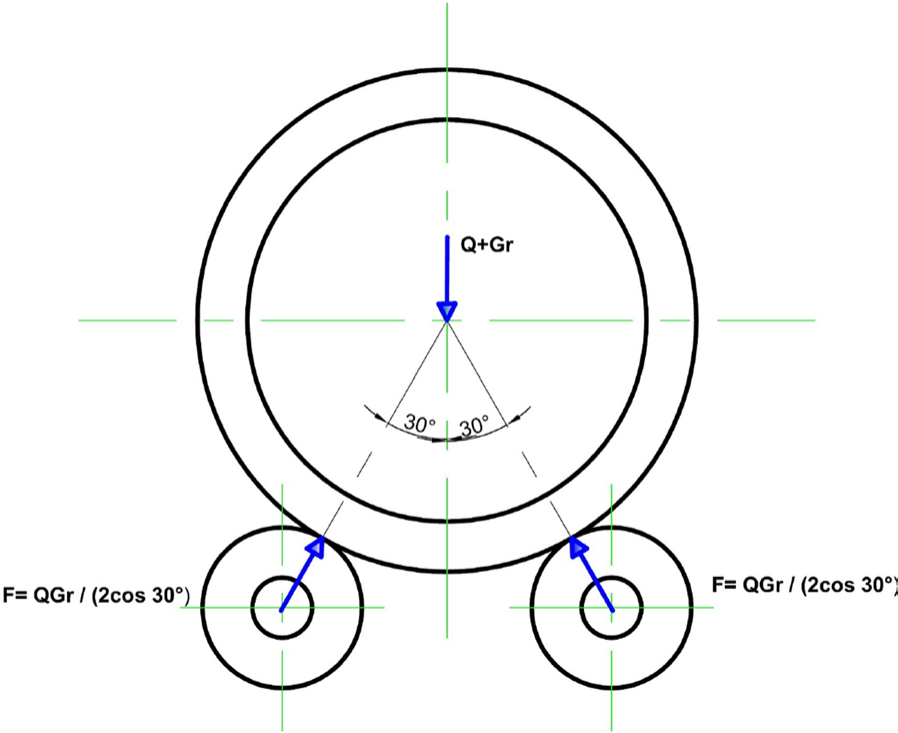
Roller station with reaction

The estimated load applied at the nodal points of the surface of the roller has been established at the nodes near the contact area between the roller and the tire. The method adopted to calculate the load distribution on the product specified is shown in Fig. 5. Equation (1) is used that can be written as (Li and Papalambros 1985):

1 $$F = \frac{Q \cdot Gr}{{2 \cdot \cos 30{^\circ }}}$$

For analyzing the reliability of the new roller shaft, it is necessary to take into account the following considerations:

The maximum reaction on the two support rollers is equal = 6454 Kn; The Tire weight is 680Kn (Deshpande and Dhekhane 2014). The load applied by roller is F = 4118,9376 Kn.

#### Data and hypothesis for analysis

**Fig. 6:**
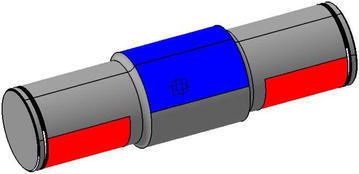
Geometry of the roller shaft
- The roller shaft material is forged steel with the technical specification (EN8), ρ = 7850 kg/m3, E = 2.05E5 MPa, ν = 0.3, Re = 600 MPa, the properties of EN8 are as showcased in Table 1 (Vijayan and Makeshkumar 2012).

- Construction drawing of the roller shaft—Fig. 6 (Sumesh Krishnan 2014).

**Table 1:** EN8 material properties (Vijayan and Makeshkumar 2012)

	Percentage of composition %					
	C	Si	Mn	S	P	Fe
EN8 material	0.40	0.25	0.80	0.015	0.015	98.52
- Angle between tire and roller is 30°.
- Load component acting on the single roller.
- We have assumed that the contact between roller and tire raceway is continuous on the total available width of ring raceway (Sumesh Krishnan 2014).
- We have assumed there is no hidden crank of shell and no large misalignment of support system axis.
- It is difficult to determine the pressure of each roller which depends on the manufacturing and assembly quality (Sumesh Krishnan 2014).
- The complexity of an entire rotary kiln and its interaction with the other components lead us to adopt some simplifying assumptions to develop our finite element model (EF).
- The kiln shell is equipped with bearings and can be considered as simply supported beam. Under normal conditions, the inlet and the outlet of the furnace do not support the shell, therefore the furnace can be taken as a cantilevered beam.
- To calculate the support reactions, a simplified procedure is used, and certain hypotheses are made: the mixture is distributed symmetrically around the vertical axis of the kiln. This means that load weight of material are evenly distributed over each roller. Figure 7 highlights the uneven distribution of load between the rollers due to inertia effects (Sumesh Krishnan 2014; Del Coz Diaz and Rodriguez Mazon 2002).

**Fig. 7:**
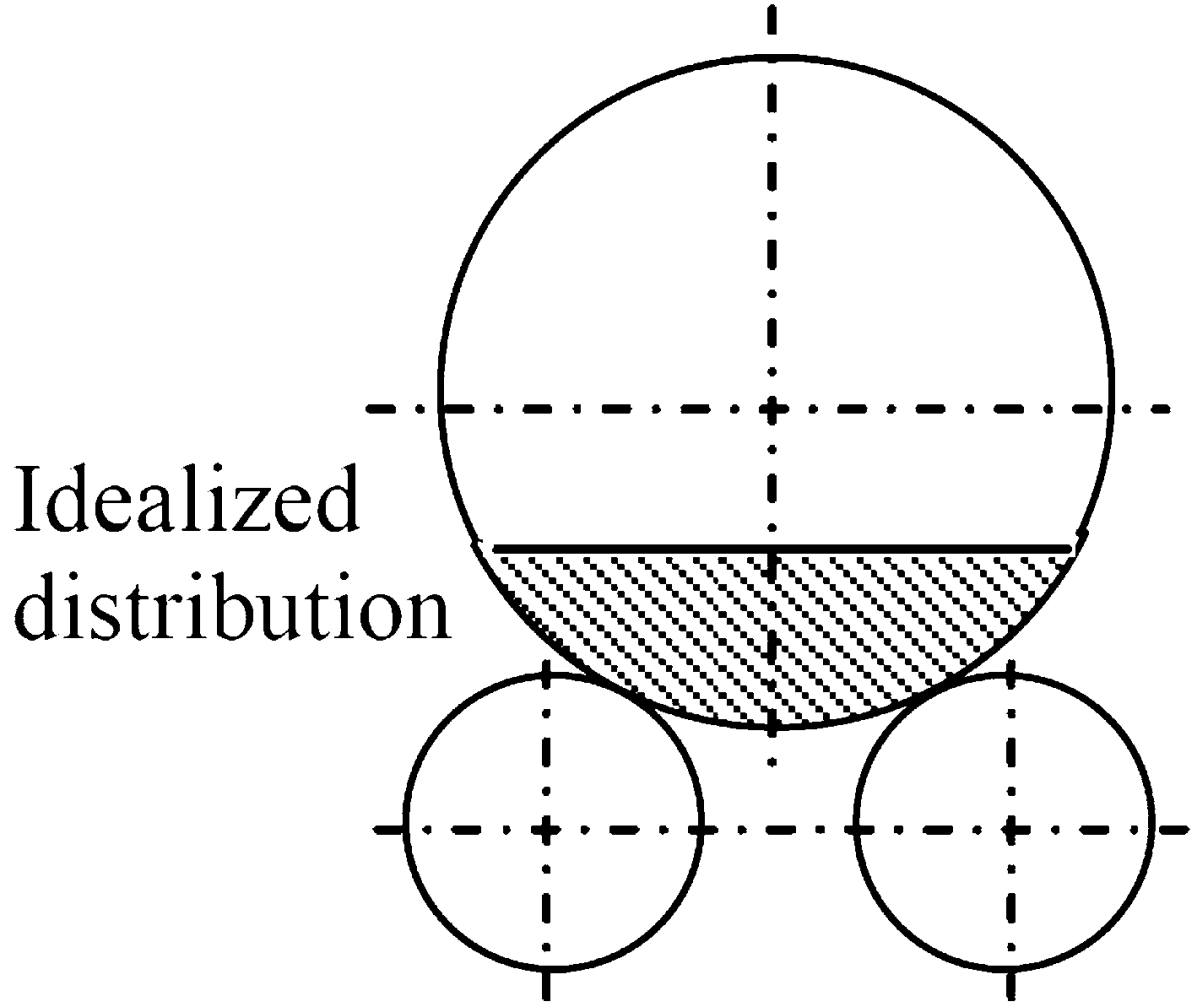
Distribution of raw-mix around vertical axis (Žiga et al. 2008).

### A finite element model

#### Boundary conditions of materials

**Fig. 8:**
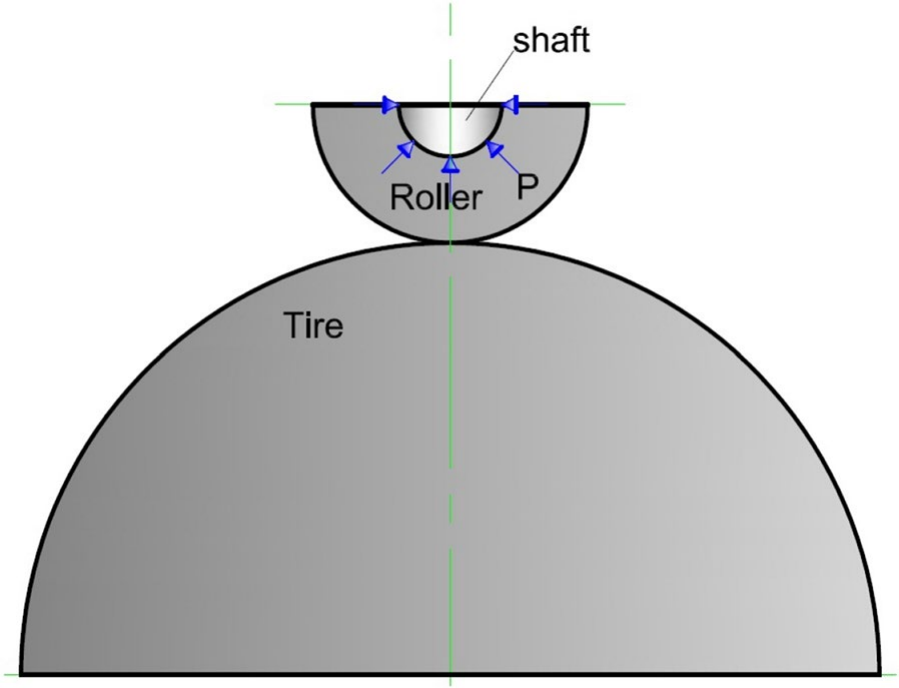
Contact between the tire and roller-simplified model (Žiga et al. 2008)

The roller and the shaft are assembled rigidly. We can consider, in the FE model, that all points shaft of the roller are in contact with the rails, are presented by the area colored red in Fig. 8, have two degrees of freedom (Sumesh Krishnan 2014).

The weight of the oven applied on the shaft of efforts rated for attachment to the seat of the roller, Fig. 8. The estimate static forces applied to the shaft was made taking into account the above data:

#### Mechanical model and mesh

The model was developed using a finite element program. We use an elastic model in large displacements. We mesh the roller shaft with volume elements 228,880. The elements used are from the library of standard components. These are quadratic elements with reduced integration for hexahedra called C3D20R and without reduced integration for tetrahedral elements called C3D10. These elements were chosen primarily for their performance in dynamic simulation (Sumesh Krishnan 2014; Del Coz Diaz and Rodriguez Mazon 2002).

### Results of static FEA

**Fig. 9:**
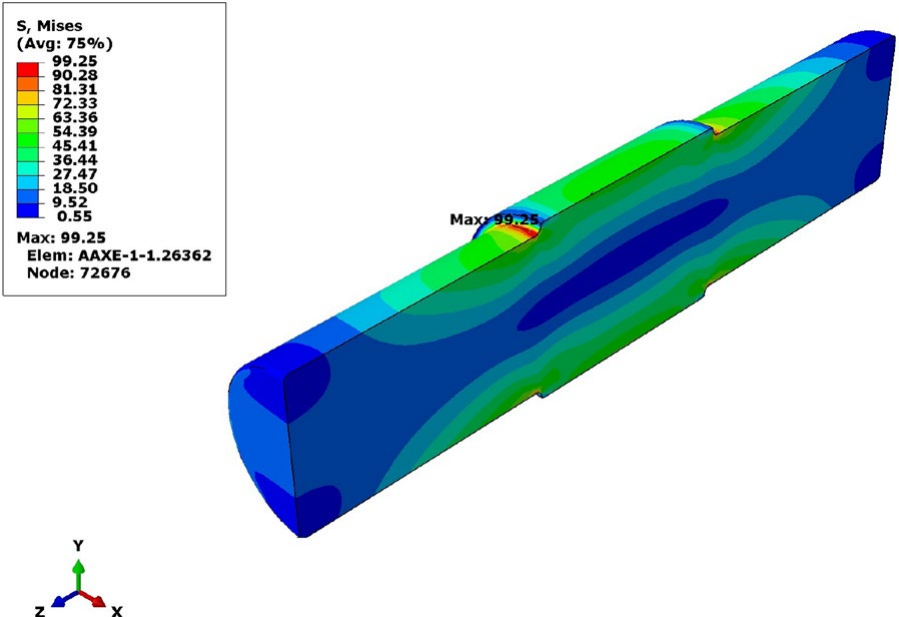
Distribution of Von Mises stresses in solid shaft

**Fig. 10:**
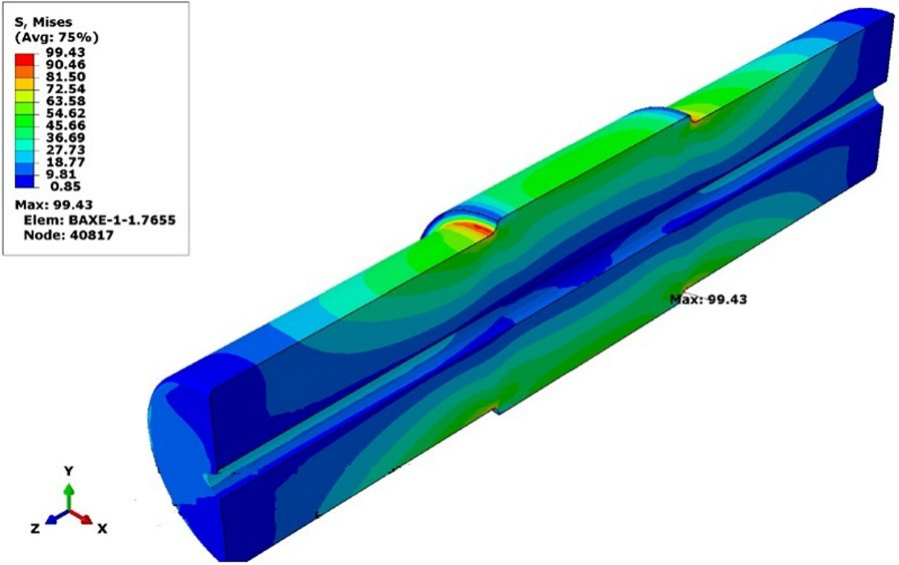
Distribution of Von Mises stresses in hollow shaft

The Von Mises stress level in the hollow shaft and the solid shaft, given by the model developed in the previous section, is shown in Figs. 9 and 10.

We observe that the most intense equivalent stresses are concentrated around the diameter of the change area. The maximum value of the stresses of the hollow shaft is increased, relative to the solid shaft, a very tolerable quantity. The maximum value of von Mises stress of 99.25 MPa is the solid shaft and 99.43 MPa for the hollow shaft.

**Fig. 11:**
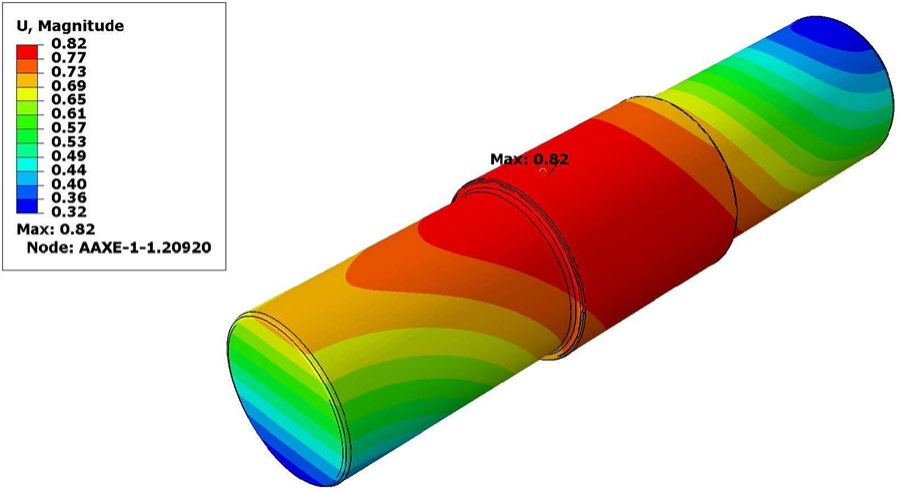
Deflection in solid shaft

**Fig. 12:**
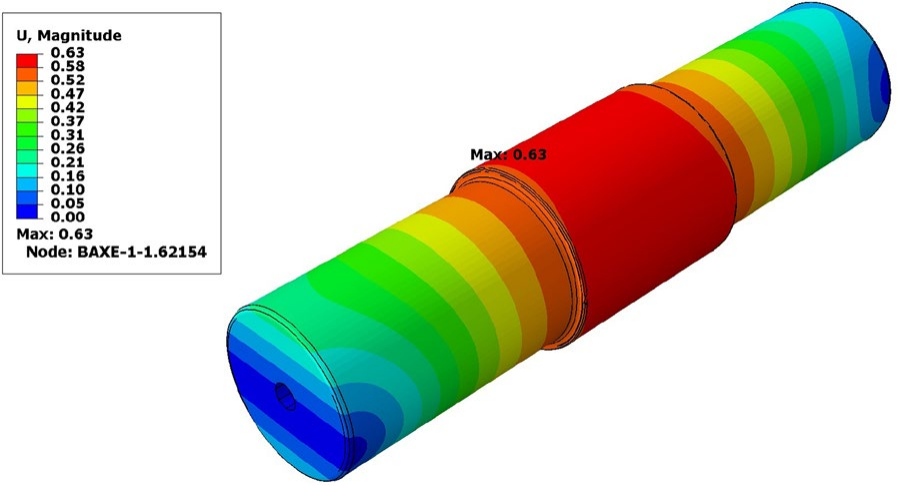
Deflection in hollow shaft

We also observe that the total displacement of the hollow shaft is 0,63 mm, which is lower than the maximum value of the displacement of the solid shaft 0.82 mm (Figs. 11, 12).

**Table 2:** Result summary

	S. Mises stress (Mpa)	Deflection (mm)
Solid shaft	99.25	0.82
Hollow shaft	99.42	0.63

#### Discussion of results

By the results (Table 2), it has become clear that the change in design of the roller shaft gives the same constraints margin constraint which confirms the validity solution without jeopardizing rigidity. It is also highlighted that changing the design of the proposed shaft, gives sufficient strength to support the load in relation to the initial design of the shaft.

The maximum permissible service life of the roller shaft can be achieved through design adopted for the hollow shaft. Therefore, while taking into account the associated external factors has been made easier accessibility significantly to the roller shaft during preventive maintenance.

## Development of an ultrasonic preventive control method of roller shaft

### Method principle

**Fig. 13:**
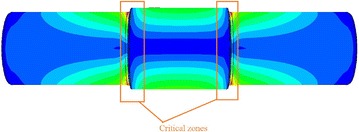
Critical zones on roller shaft

In order to determine whether there are defects in the part (roller shaft), particularly in the critical zones identified Fig. 13, we proceeded to calibrate the ultrasonic tester and build a support sensor having a rod having a scale to determine and locate the fault position and manufacture shims for maintaining the position of the sensor relative to the surface of the shaft hole (Norme française 2008).

**Fig. 14:**
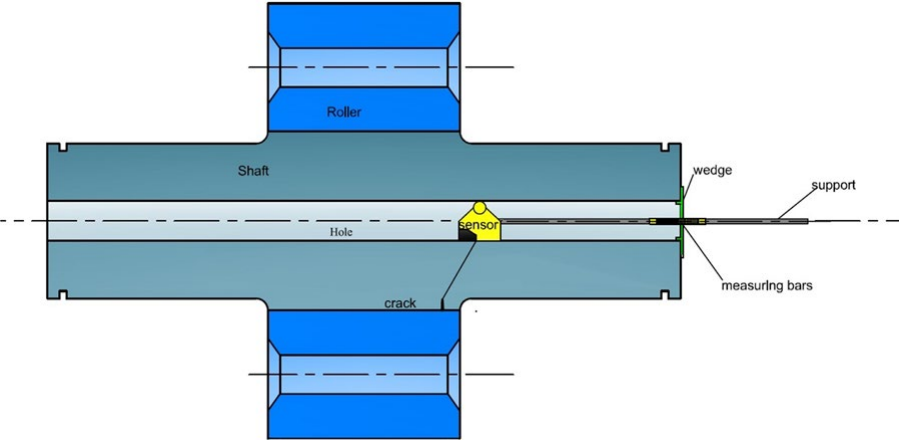
Ultrasonic nondestructive testing shaft

Transverse waves are used to control the critical zones shaft in order to achieve control without dismantling the covers, thrust collar and the oil change for the six rollers and also for their assembly. The transverse ultrasonic waves are generated by a sensor, with a definite angle from the surface of the shaft hole. As shown in the diagram in Fig. 14. This method allows the controller to scan the entire Critical Zone by transverse waves.

The surface of the shaft being circular, it is necessary to control a rotation of a turn (360°) to ensure scanning of controlled set. Also, we will develop a rotary system to support the sensor, which will allow a dramatically shortening the time (Garayoa et al. 2002).

The hollow shaft inspection system includes a mechanical subsystem, that positions the ultrasonic transducers against the shaft bore inner surface and performs scanning along the shaft bore, The transducer (see Fig. 14) required for hollow shaft inspection are mounted on a cylindrical holder, which performs the axial and angular scan movements of the transducer head. Transducers are mounted by pairs on spring-loaded shoes that guarantee correct transducer to shaft surface contact.

The mobility and flexibility of the system allows to inspect any type of hollow roller shaft on kiln using specifically designed probe system modules and roller shaft adapter. The roller shaft control system aims faster, cheaper, reliable and easier.

### Calibration procedure

**Fig. 15:**
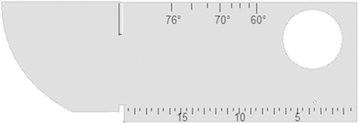
Standardized calibration block V1

Section 7.6 of ISO 9001 (Norme française 2008) requires the Control of monitoring and measuring equipment, among others it is specified regular calibration of monitoring equipment, which is not always easy. Indeed, during the calibration of the device with the normalized standard (Fig. 15) it is important to know equipment reliability: testers, sensors (control sensors) and more generally the conditions for checking.

So a first step we do a calibration according to the standard of the non-destructive ultrasonic testing in art, using a standard gauge block V1 shall conform to EN 12223, as shown in Fig. 15.

The first three of the following operations on first step are essential because the probe index and the beam spread must be found by practical measurement and not assumed to be correct from probe markings and theoretical considerations.

#### Determination of probe index

**Fig. 16:**
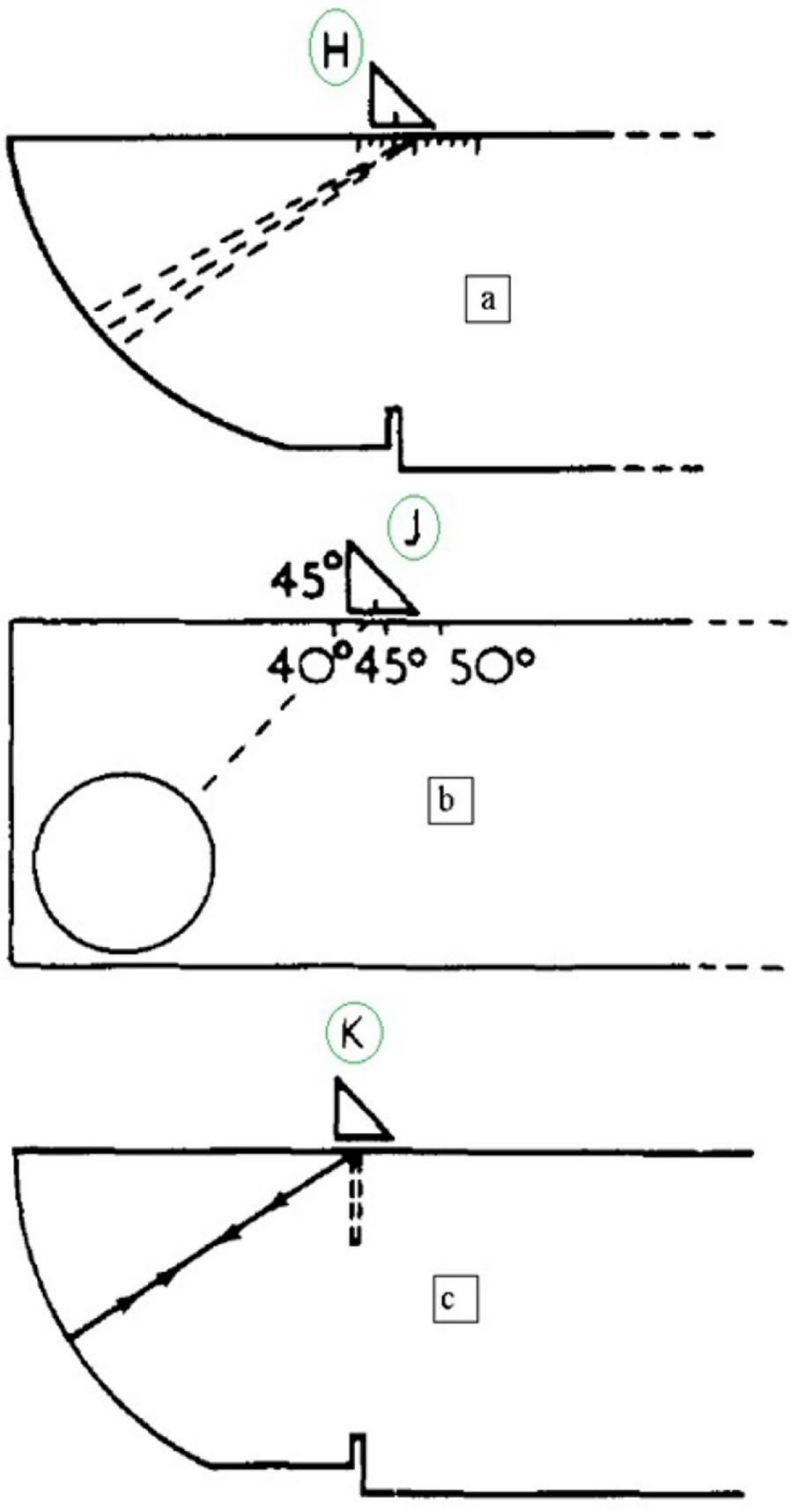
**a** Probe H in position to determine probe index. **b** Probe J in position to determine the angle of refraction. **c** Position probe at K to find the zero point of the trace for a shear wave. 50 subdivisions on the screen represent 100 mm of steel thickness

The probe index is given on the probe; in order to check that the position marked on the probe is correct, place the probe in position H (Fig. 16a). Move the probe until maximum amplitude is received from the 100 mm curved surface. The central mark on the graduated scale will be the position at which the beam leaves the Plexiglass and enters the steel, i.e. the probe index.

#### Determination of the angle of refraction

Move the probe until maximum signal amplitude is obtained from the Plexiglass cylinder (Fig. 16b). The reference block has calibrated scales engraved at 40°–70° and the relevant angle of refraction marked on the probe should coincide with the correct scale position. The probe index which has been previously determined should be marked on the probe in order to obtain the correct results.

#### Correction of the zero point

The presence of the Plexiglass in the transverse wave probe causes a time lag between the moment at which the signal leaves the transducer and the moment it leaves the wedge. This time lag must be corrected for and the zero point set on the cathode ray screen. Obtain an echo at a maximum amplitude on the screen from 100 mm radius (Fig. 16c). Adjust the echo by movement of the horizontal shift and the fine time base control in order to obtain two echoes on the screen at 50 subdivisions and 100 subdivisions; the zero on the scale will then correspond to the moment the beam leaves the Plexiglass wedge.

After initial calibration, we will calibrate the sensitivity of the apparatus, on which one will be used for line control of the service shaft.

Specifically designed Reference blocks of roller shaft hollow shall be made available when sensitivity is to be established by the distance amplitude curve (DAC) technique, when defects are to be sized in terms of amplitude relative to reference reflectors by the DAC technique. The surface condition of the reference block shall be representative of the surface condition of the part to be examined. Unless otherwise specified the reference block shall contain at least three reflectors covering the entire depth range under examination (Lavender 1976).

The angle beam shall be directed toward the reference reflector (holes or notches) that yields the maximum response in the area of interest. The gain control shall be set so that this response is 80 % ± 5 % of full screen height. This shall be the reference level. The search unit shall then be manipulated, without changing instrument settings, to obtain the maximum responses from the other calibration reflectors at their beam paths to generate the distance amplitude correction curve (DAC).

The test sensitivity requires consideration. As mentioned above, the sensitivity level adopted must assure detection of small cracks in the early stages of growth, whilst not causing the over amplification of insignificant signals which would result in the rejection of good components.

New inspection system provides information on the position of the defects on their relative importance compared to artificial defects (DAC) and therefore a classification to know the severity of these defects (Norme française 2000).

## Conclusion

There are many external causes of failure of the shaft that can be eliminated by changing the entire design of the oven, something that is not possible, very expensive and time consuming. Instead of changing the design of the whole; it would be better and simpler to change only the design of the roller shaft as described in our article, adopting a hollow shaft.

The new design facilitates control by enabling access to critical areas and preventive maintenance. In addition, it results in a saving of time, reducing downtime for maintenance and weight gain of the material constituting the roller shaft (hollow shaft).

Therefore, adoption of the change in the outlook and physical design is a factor taking into account the recommended conditions and parameters considered during maintenance.

In perspective, it is proposed the integration of the control system to the roller shaft, allowing continuous monitoring of the shaft. Translators can be integrated into the hole of the roller shaft, which will monitor the critical areas that we have identified above. A study is underway to achieve this.
